# Quercetagetin and Patuletin: Antiproliferative, Necrotic and Apoptotic Activity in Tumor Cell Lines

**DOI:** 10.3390/molecules23102579

**Published:** 2018-10-09

**Authors:** Jesús J. Alvarado-Sansininea, Luis Sánchez-Sánchez, Hugo López-Muñoz, María L. Escobar, Fernando Flores-Guzmán, Rosario Tavera-Hernández, Manuel Jiménez-Estrada

**Affiliations:** 1 Laboratorio 2-10, Departamento de Productos Naturales, Instituto de Química, Universidad Nacional Autónoma de México, 04510 Ciudad de México, Mexico; javier33@comunidad.unam.mx (J.J.A.-S.); rosario.tavera@gmail.com (R.T.-H.); 2 Laboratorio 6, 2do piso, UMIEZ, Facultad de Estudios Superiores Zaragoza, Universidad Nacional Autónoma de México, 09230 Ciudad de México, Mexico; luisss@unam.mx (L.S.-S.); hugolm@comunidad.unam.mx (H.L.-M.); molekulare@yahoo.com.mx (F.F.-G.); 3 Laboratorio de Microscopía Electrónica, Departamento de Biología Celular, Facultad de Ciencias, Universidad Nacional Autónoma de México, 04510 Ciudad de México, Mexico; escobarluisa@ciencias.unam.mx

**Keywords:** necrotic, apoptosis, quercetin, quercetagetin, patuletin

## Abstract

Quercetagetin and patuletin were extracted by the same method from two different *Tagetes* species that have multiple uses in folk medicine in Mexico and around the globe, one of which is as an anticancer agent. Their biological activity (IC_50_ and necrotic, apoptotic and selective activities of these flavonols) was evaluated and compared to that of quercetin, examining specifically the effects of C6 substitution among quercetin, quercetagetin and patuletin. We find that the presence of a methoxyl group in C6 enhances their potency.

## 1. Introduction

Cancer is one of the most severe public health problems worldwide. The urgent need to obtain new drugs to treat it arises from the fact that conventional treatments cause many side effects that negatively impact patients’ quality of life. This has fostered the use of plant extracts and their metabolites as sources of active compounds [1]. Plants are still widely-used for medicinal purposes by the indigenous peoples in Mexico, and the biological activity of many plant derivatives has been evaluated and shown to have medicinal importance [2]. The genus *Tagetes* belongs to the family Asteraceae. It is highly-esteemed as an ornamental plant, and in some parts of the world is has ritual-cultural importance [3]. Several species of *Tagetes* are found in Mexico, some known commonly as Cempasuchil or Cempoalxochitl [4]. Two representative species are *Tagetes erecta* and *Tagetes patula* [5], both of which have important therapeutic potential due to their antiparasitic, antimalarial, anti-inflammatory, antitumorogenic and antiviral effects [6].

*Tagetes* flowers contain different classes of natural products [7], including terpenes, carotenoids and flavonoids, which are known to have pharmacological effects [8]. Differences in their molecular structures may be responsible for their different pharmacological properties [9]. Specifically, the genus *Tagetes* has many kinds of flavonoids [10]. Of these, quercetagetin and patuletin present distinct inhibitory activity [11]. In the present study, we implemented a method to obtain quercetagetin and patuletin from *Tagetes erecta* and *Tagetes patula*, respectively [12,13], and performed a structure-activity relationship (SAR) study [14], focused on their anticancer properties by comparing them to quercetin [15].

**Figure 1:**
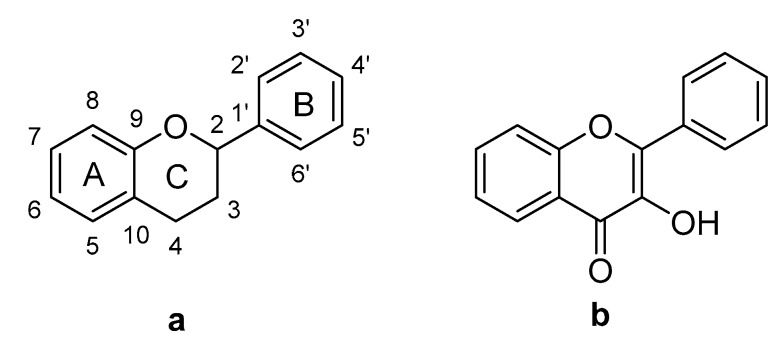
(**a**) Base structure of flavonoids; (**b**) Structure of flavonols.

Flavonoids are polyphenolic compounds with a C_6_-C_3_-C_6_ phenylbenzopyrone structure (Figure 1a) and they exhibit great structural diversity due to their various hydroxylation, methoxylation, glycosylation and acylation patterns [16]. The basic structure of the flavonoid nucleus has different substitution patterns in the A, B and C rings that result in various subgroups, one of which are the flavonols, which possess a 3-hydroxyflavone structure (Figure 1b). Examples of this type of flavonoid are the compounds quercetin, myricetin and kaempferol [17]. Flavonoids display diverse biological activities, including chemopreventive and chemotherapeutic effects on cancer. The following mechanisms of action have been reported: carcinogen inactivation, antiproliferation, cell-cycle arrest, induction of apoptosis, inhibition of angiogenic processes, modulation of multi-drug resistance, and antioxidative activity [18,19].

**Figure 2:**
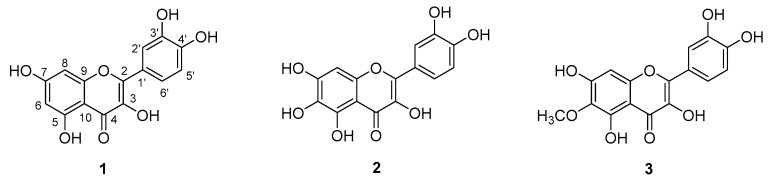
Structures of (**1**) quercetin, (**2**) quercetagetin and (**3**) patuletin.

Some structure-activity relationship studies have been conducted, and they indicate that the presence of the C2=C3 double bond and the hydroxylation pattern of flavonoids influence tumor proliferation modulation [20]. In this regard, research has shown that hydroxylated flavonoids possess stronger inhibitory activity on cancer cells than permethoxylated flavonoids, and that C3-hydroxylation is important for this activity. Apigenin, for example, which lacks a 3-OH group, presents lower antiproliferative activity than kaempferol [21]. The catechol group in ring B enhances anticancer activity [22]. There are few studies of substituents at C6 in relation to anticancer activity, but flavonols like quercetin, quercetagetin and patuletin all have the aforementioned structural characteristics that seem to be important for antiproliferative activity. The only difference among these compounds is the C6 group substituent, as quercetin has a C6-H, quercetagetin has a C6-OH, and patuletin has a C6-OMe (Figure 2). Given this background, the present study was designed to contribute to the study of the role of C6 substitution in ring A in the cytotoxic activity of flavonols like quercetin, quercetagetin and patuletin. The fact that some cancer cells show resistance to certain kinds of cell death can pose a problem for treatment success, so identifying and characterizing molecules that induce apoptosis or necrosis [23] will allow us to add new strategies to existing therapeutic approaches.

## 2. Results

In the present study, quercetagetin (**2**) and patuletin (**3**) were isolated by the same method from different species of *Tagetes* plants. Upon comparing the structure of isolated compound **2** and quercetin, we found that they share a basic flavonol structure type and that the only difference is the substituent group at the ring-A C6 position (Figure 2). These structures were confirmed by 1D and 2D NMR experiments that assigned the quercetagetin hydrogen and carbon positions.

### 2.1. Identification of Compounds ***2*** and ***3***

**Figure 3:**
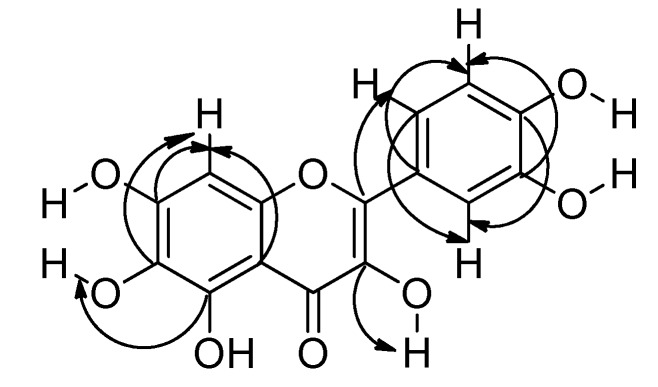
HMBC correlations of quercetagetin.

Compound **2** was isolated as a yellow powder. The structure was elucidated and the compound identified as quercetagetin by 700 MHz 1D and 2D NMR experiments in DMSO-*d*_6_. The ^1^H-NMR spectrum displayed six hydroxyl proton signals and four aromatic proton signals at δ_H_ 7.66 (1H, d, *J* = 2.2 Hz, H-2′), 7.53 (1H, dd, *J* = 8.5, 2.2 Hz, H-6′), 6.88 (1H, d, *J* = 8.5 Hz, H-5′) and 6.49 (1H, s, H-8). This assignment disagrees with a previously reported one [13] because the HSQC experiment showed correlations between the protons mentioned above with the aromatic carbons at δ_c_ 115.05 (C-2′), 119.91 (C-6′), 115.03 (C-5′) and 93.22 (C-8). Additionally, the ^13^C-NMR spectrum showed 15 signals corresponding to the base structure of flavonols and a signal at δ_c_ 175.84 corresponding to a carbonyl group (C-4). The other carbon atom assignments were made with the support of HMBC experiments (see Supplementary Material) and the corresponding correlations are shown in Figure 3. Complete assignments are listed in Table 1.

The molecular formula of **2** was verified by HR-DART-MS as C_15_H_11_O_8_ by an [M + H]^+^ ion peak at *m*/*z* 319.04553, indicating the molecular formula was C_15_H_10_O_8_. Compound **3** was isolated as a yellow powder and identified as patuletin. The HR-DART-MS of **3** showed a ion peak at *m*/*z* 333.6114 [M + H]^+^ (calcd. for C_16_H_13_O_8_: 333.06104) indicating the molecular formula was C_16_H_12_O_8_. The structure was elucidated by 1D and 2D NMR experiments carried out at 700 MHz in CD_3_OD, and compared with the structure reported in [11] the displacements were very similar, however, the values of the quaternary carbons at δ_c_ 158.46, 153.61 and 153.00 could be interchangeable due to their proximity and the last two only correlate with the proton H-8 (δ_H_ 6.50). The ^1^H-NMR spectrum displayed four aromatic protons signals at δ_H_ 7.74 (1H, d, *J* = 2.2 Hz, H-5′), 7.64 (1H, dd, *J* = 8.5, 2.2 Hz, H-6′), 6.89 (1H, d, *J* = 8.5 Hz, H-2′) and 6.50 (1H, s, H-8), methyl connected to an oxygen protons were displayed at δ_H_ 3.88 (OCH_3_, s, 3H). The assignments of the carbons and protons of patuletin in comparison with NMR literature data are given in Table 1.

### 2.2. Antiproliferative Activity

**Figure 4:**
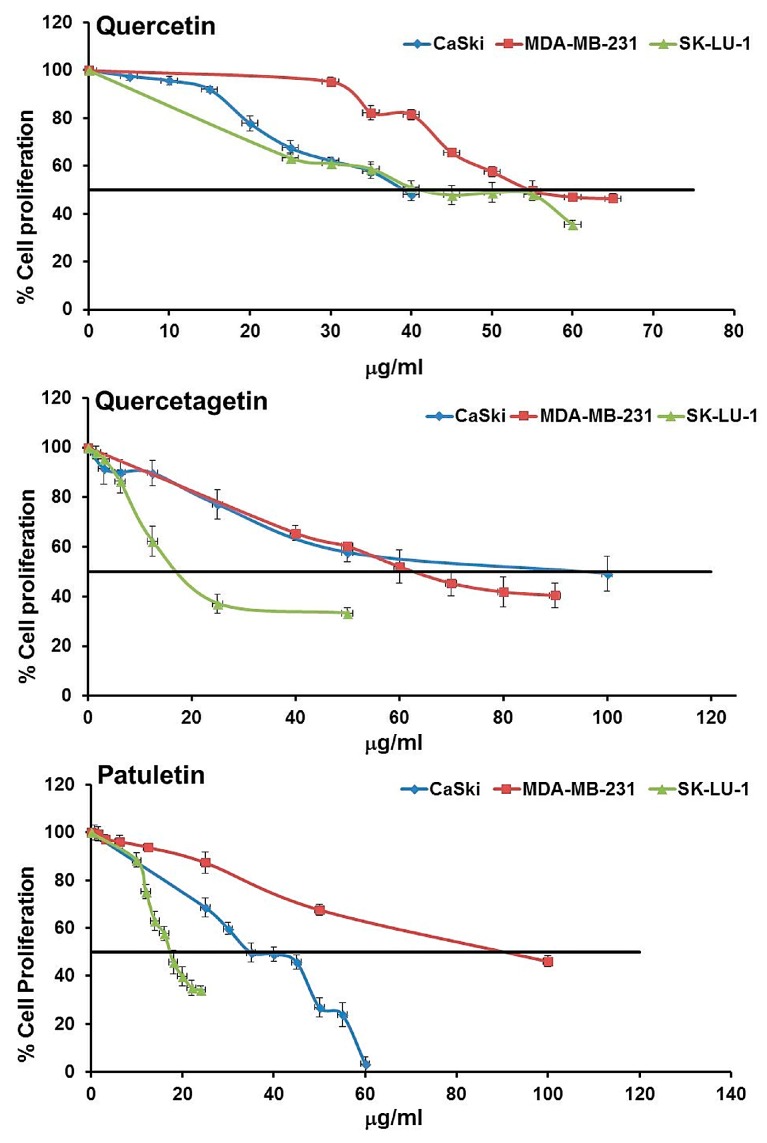
Dose-response curves of the antiproliferative effect of quercetin, quercetagetin and patuletin.

To determine the concentration of flavonols required to inhibit the proliferation of CaSki, MDA-MB-231 and SK-Lu-1 by half (IC_50_), 7500 cells were cultured for 24 h with 6, 12, 25, 50 and 100 μg/mL of quercetin, quercetagetin or patuletin. After 24 h, the number of cells was evaluated using crystal violet staining (Figure 4, Table 2).

Results showed a dose-dependent proliferation inhibition (Table 2). The IC_50_ values calculated were between 37 and 88 μg/mL (Table 2). It is interesting to note that the three cell lines showed different susceptibilities. The IC_50_ values of the three compounds for the SK-Lu-1 cell line suggest that they could be more effective for this particular type of cancer.

### 2.3. Necrotic Activity in Tumor Cells

**Figure 5:**
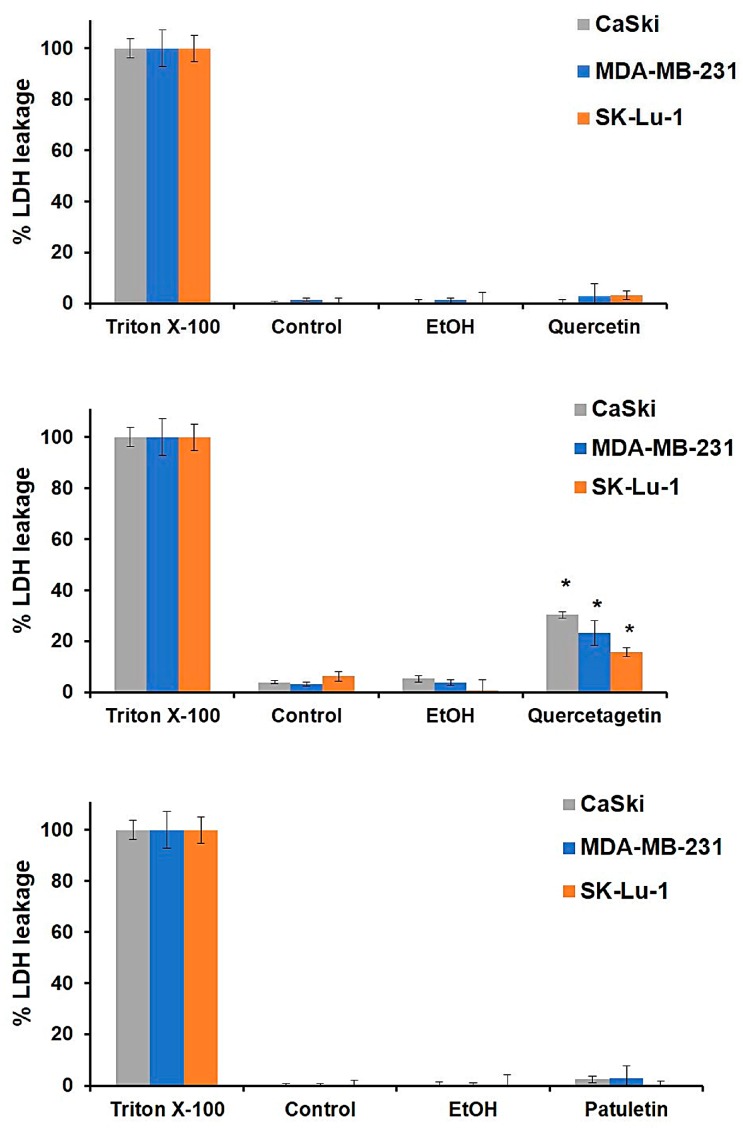
LDH activity induced by quercetin, quercetagetin and patuletin on CaSki, MDA-MB-231 and SK-Lu-1 cultures. In each case, 7500 cells/well were seeded in 96-well tissue culture plates. After 24 h, the medium was removed, and the cells were stimulated with the IC_50_ values for quercetin, quercetagetin and patuletin, or EtOH (10 μL/mL). The amount of lactate dehydrogenase (LDH) released into the culture supernatant was measured after 24 h of treatment. Experimental data are presented as mean ± S.D. for three independent experiments with three repetitions. * *p* < 0.05 versus EtOH (ANOVA followed by a Tukey’s test).

The necrotic activity of quercetin, quercetagetin and patuletin was evaluated to determine whether they induce necrosis (Figure 5). CaSki, MDA-MB-231 and SK-Lu-1 cultures were exposed to the compounds at the aforementioned IC_50_ values, and the amount of LDH released into the culture supernatant after treatment was used as a measure of the loss of plasma-membrane integrity. In addition, the three cancer cell lines were treated with Triton X-100 in independent experiments, and the LDH released was adjusted to 100% to serve as a positive control. Our results show that quercetin and patuletin do not manifest necrotic cell death activity, as evidenced by the fact that the cellular decrease observed in the treated cultures was not a consequence of a necrotic process. This finding suggests that some other type of cell death, distinct from necrosis, was responsible for the decrease in the number of cells. In contrast, the amount of LDH released in the cells treated with quercetagetin did indicate significant necrotic activity (15–30%), suggesting that part of the decrease in the number of cells using this compound was due to a necrotic process. As a result, we would suggest that the structure of quercetagetin, which has a hydroxyl group in C6, could be important for this compound’s necrotic activity. It is important to mention that there are no existing reports of necrotic activity in relation to any of these three molecules.

### 2.4. Apoptotic Bodies under DAPI Staining

**Figure 6:**
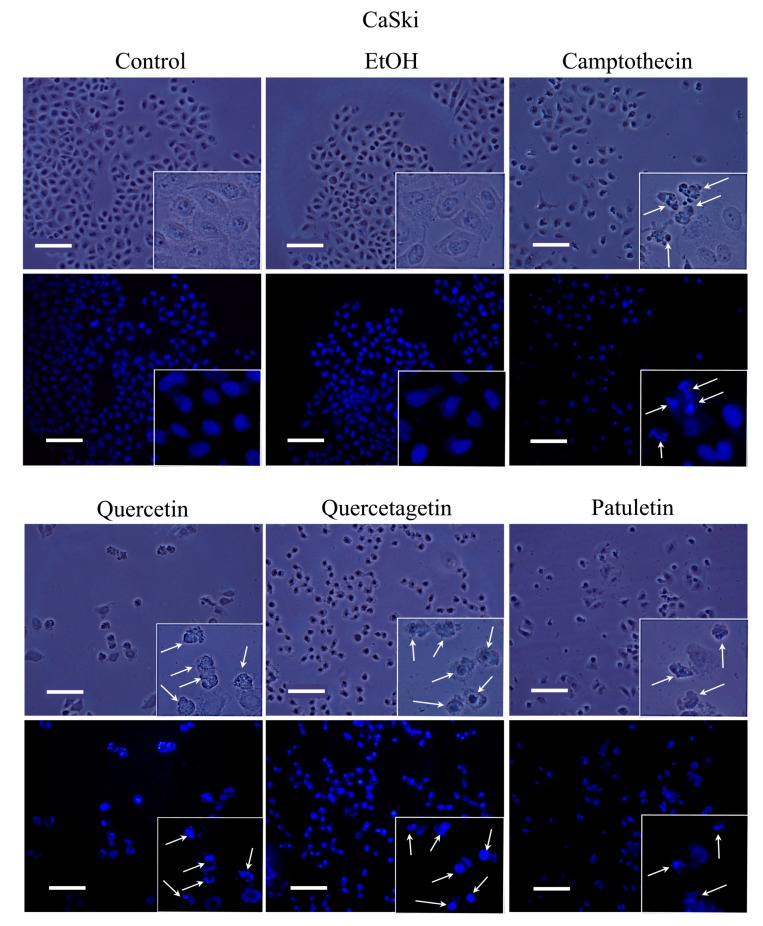
Morphology and DAPI staining. CaSki cell cultures were exposed to quercetin, quercetagetin and patuletin at the aforementioned IC_50_ values. Camptothecin was used as a positive control for apoptosis. The phase-contrast images evidenced cellular shapes under the different experimental conditions. The control and EtOH cells had extended cytoplasm with chromatin distributed in the nuclear space, as shown by the fluorescent distribution of DAPI. After treatment, both the cytoplasm and nucleus were contracted, and several apoptotic bodies were visible (arrows), Scale bars 100 microns.

**Figure 7:**
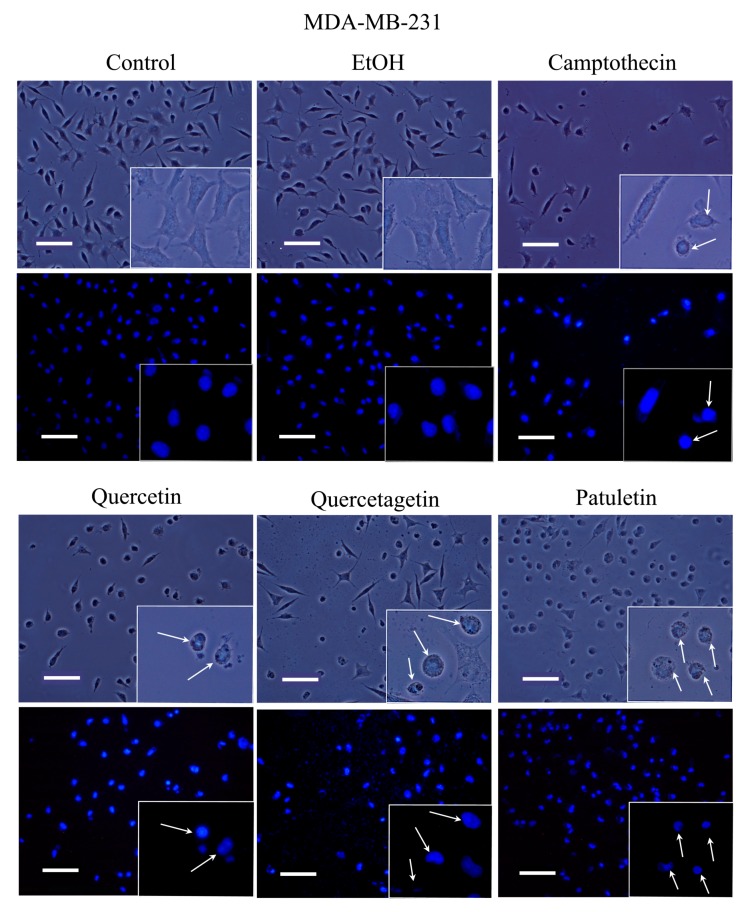
Morphology and DAPI staining. MDA-MB-231 cell cultures were exposed to quercetin, quercetagetin and patuletin at the aforementioned IC_50_ values. Camptothecin was used as a positive control for apoptosis. The phase-contrast images evidenced cellular shapes under the different experimental conditions. The control and EtOH cells had extended cytoplasm with chromatin distributed in the nuclear space, as shown by the fluorescent distribution of DAPI. After treatment, both the cytoplasm and nucleus were contracted, and several apoptotic bodies were visible (arrows), Scale bars 100 microns.

**Figure 8:**
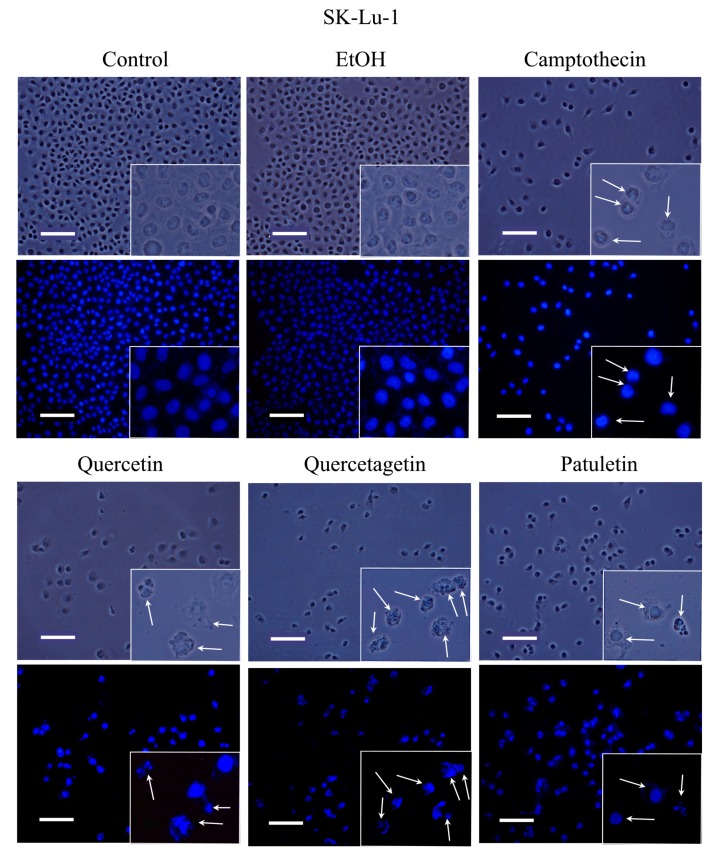
Morphology and DAPI staining. view of SK-Lu-1 cultures, were exposed to quercetin, quercetagetin and patuletin at the aforementioned IC_50_ values. Camptothecin was used as a positive control for apoptosis. The phase-contrast images evidence cellular shapes under the different experimental conditions. The control and EtOH cells had extended cytoplasm with chromatin distributed in the nuclear space, as shown by the fluorescent distribution of DAPI. After treatment, both the cytoplasm and nucleus were contracted, and several apoptotic bodies were visible (arrows), Scale bars 100 microns.

Apoptosis is characterized by morphological changes and chromatin condensation that produce smaller, more compact nuclei and/or the formation of apoptotic bodies. Our CaSki, MDA-MB-231 and SK-Lu-1 cell cultures were stimulated with quercetin, quercetagetin and patuletin to evaluate morphological changes, chromatin condensation and the formation of apoptotic bodies. The latter were identified by staining with fluorochrome 4,6-diamidino-2-phenylindole (DAPI) and epifluorescence microscopy observation (Figure 6, Figure 7 and Figure 8).

Observations under phase-contrast illumination revealed that the morphology of the control and vehicle cells was conserved, with a polyhedral shape and extended cytoplasm. In these conditions, chromatin is distributed throughout the nucleoplasm (see DAPI staining). After treatment with quercetin, quercetagetin or patuletin, the morphology of the cells was clearly altered, as they showed shrinkage, evidenced by the loss of the polyhedral form. Compact nuclei and apoptotic bodies were also visible, suggesting the presence of the apoptotic process in the CaSki (Figure 6), MDA-MB-231 (Figure 7), and SK-Lu-1 (Figure 8), cells. Despite these results, it is still necessary to detect the activation of effector caspases such as active caspase-3, to establish that a caspase-dependent apoptotic process has been activated. Thus, more conclusive experiments were performed, as described below.

#### Detection of Active Caspase-3, Active Caspase-8, Active Caspase-9 and PARP

The caspases are a family of cysteine proteases that function as crucial mediators of apoptosis. Poly (ADP-ribose) polymerases (PARPs) are a family of related enzymes that play an important role in DNA repair. PARP is a cleavage that occurs during increased levels of active caspase-3, which is likely the most well-understood of the mammalian caspases in terms of its specificity and role in apoptosis. Caspase-3 is also required for some typical hallmarks of apoptosis and is indispensable for apoptotic chromatin condensation and DNA fragmentation. Likewise, it is essential for certain processes associated with the formation of apoptotic bodies. The apoptosis process can be conducted in two classic signaling pathways: one extrinsic, the other intrinsic.

The extrinsic pathway is mediated by caspase-8, while caspase-9 is determinant in the intrinsic pathway. In this study, we identified active caspase-3 and PARP by means of western blots to determine the presence of the apoptotic process. In addition, we immunodetected active caspase-3, active caspase-8 and active caspase-9 to evaluate the presence of these enzymes in the three cell lines treated with quercetin, quercetagetin and patuletin. Finally, all of these caspases were evaluated using flow cytometry to obtain quantitative results.

**Figure 9:**
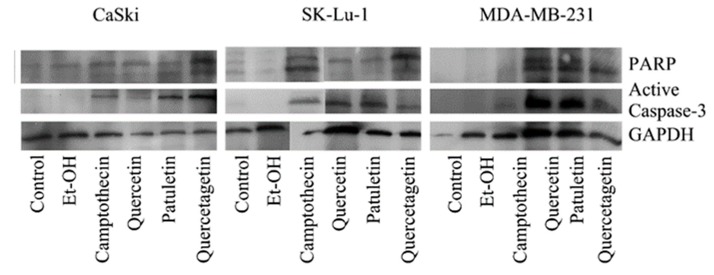
Expression of active caspase-3 and PARP in CaSki, MDA-MB-231 and SK-Lu-1 cell cultures exposed to quercetin, quercetagetin and patuletin at the aforementioned IC_50_ values.

**Figure 10:**
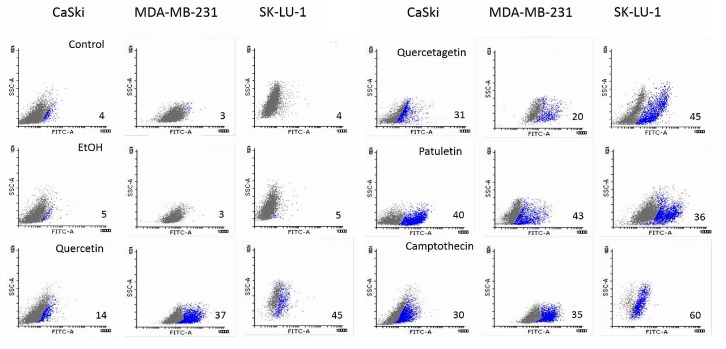
Detection of active caspase-3 in CaSki, MDA-MB-231 and SK-Lu-1 cell cultures exposed to quercetin, quercetagetin and patuletin at the aforementioned IC_50_ values. Cells were analyzed by FACS from Cell Quest; numbers indicate the percentages of caspase-3 activity.

Results show that the expression of active caspase-3 and PARP proteins occurred in all three cell lines treated with quercetin, quercetagetin and patuletin (Figure 9). This confirms the participation of the apoptotic process during elimination of cells treated with the compounds used in this study. The quantitative evaluation of active caspase-3 by cytometry (Figure 10) showed that the number of cells positive to active caspase-3 had different percentages: SK-Lu-1 cells presented greater susceptibility to quercetin and quercetagetin, since these presented a higher percentage of positive cells compared to the other two cell types (Table 3). Conversely, MDA-MB-231 proved to be more sensitive to patuletin (Table 3). Our morphological and biochemical results evidence the presence of apoptosis during elimination of the cancer cells treated with quercetin, quercetagetin and patuletin.

**Figure 11:**
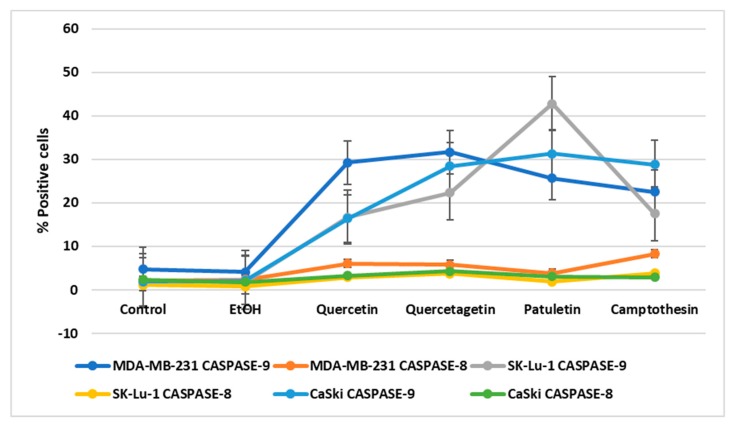
Percentage of positive cells to active caspase-8 and caspase-9 treated with quercetin, quercetagetin, and patuletin.

Active caspase-9 and active caspase-8 were also present in the treated cancer cells, though the numbers of cells positive to caspase-9 detection were significantly higher than those that were positive to caspase-8 (Figure 11). This finding suggests that quercetin, quercetagetin and patuletin induce the apoptotic process through the intrinsic apoptotic pathway (see Supplementary Material).

### 2.5. Effect of Quercetin, Quercetagetin and Patuletin on Non-Tumor Cells

The main compounds currently used in chemotherapy present problems in terms of their selective activity on malignant cells and because they produce undesirable side effects. We thus considered it crucial to determine the selectivity of the compounds tested in the present work, and to evaluate their antiproliferative activity in non-tumor cells in order to reach conclusions concerning their potential anticancer activity. Our research has demonstrated the anti-proliferative activity of quercetin, quercetagetin and patuletin in CaSki, MDA-MB-231, and SK-Lu-1 tumor cells; however, their effect on non-tumor cells has not yet been determined. To test the selectivity of the compounds evaluated, we used lymphocytic cells. A sample of an enriched lymphocyte population (ELP) from peripheral blood was treated with the three compounds to assess the proliferation of this cellular fraction. ELPs from a normal blood donor were induced for three cycles of proliferation by phyto-hemagglutinin and labeled with 5(6)-carboxyfluorescein diacetate *N*-succinimidyl ester (CFSE). These cells were treated with quercetin, quercetagetin and patuletin after 48 h of culture (second proliferation cycle) and then cultured for another 72 h. The cells were harvested, and their proliferative potential was analyzed by flow cytometry.

**Figure 12:**
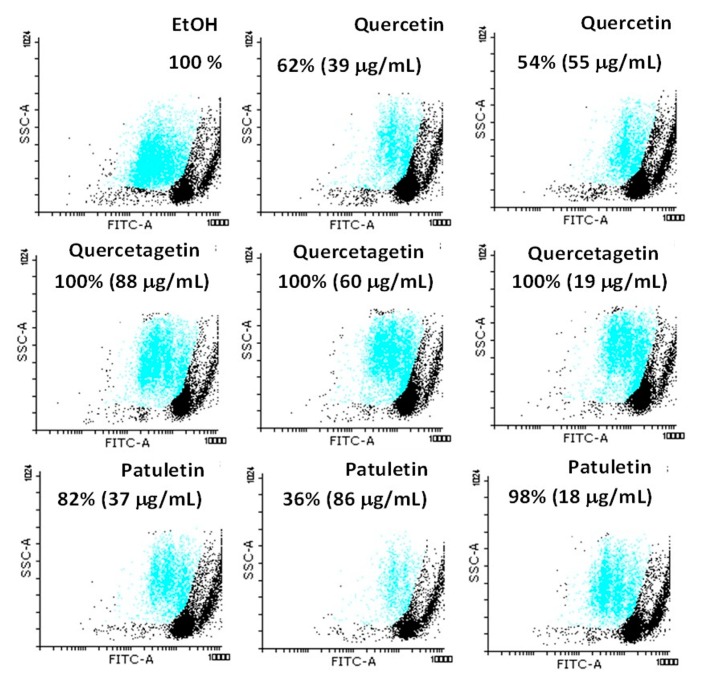
Evaluation of the anti-proliferative effect of quercetin, quercetagetin and patuletin in lymphocytes. The compounds showed no evidence of any anti-proliferative effect on normal cells.

To evaluate the anti-proliferative potential of the flavonoids on lymphocytes, cells were cultured at the IC_50_ concentration values for 24 h and then labeled with 5(6)-carboxyfluorescein diacetate *N*-succinimidyl ester (CSFE). Fluorescence was measured using a cytometer. A culture without flavonoids was used as a positive control, while eluents were used as negative controls. Normal lymphocytes were induced for two cycles of proliferation by phyto-hemagglutinin (Figure 12). Quercetin, quercetagetin and patuletin all produced significant proliferation at the concentration employed.

Cells treated only with the vehicle only were used as an experimental control (EtOH). They showed 100% proliferation (Figure 12); however, when the lymphocytes were treated at the aforementioned IC_50_ values of the compounds their behavior differed, as the proliferative potential of lymphocytes was negatively-affected by treatment with 86 μg/mL of patuletin. The treatment with quercetin affected all the cells evaluated in a proportion of approximately 50%. In contrast, quercetagetin did not affect the proliferation activity of the lymphocytes.

### 2.6. Necrotic Effect of Quercetin, Quercetagetin and Patuletin in Non-Tumor Cells

**Figure 13:**
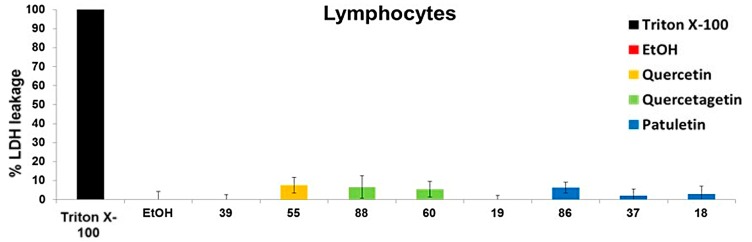
Evaluation of necrosis by quercetin, quercetagetin and patuletin on normal cell lymphocytes. The medium was removed, and the cells were stimulated to the level of IC_50_ values for quercetin, quercetagetin and patuletin, as well as with EtOH (10 μL/mL). After 24 h, the amount of lactate dehydrogenase (LDH) released from the culture supernatant was evaluated. Experimental data are presented as mean ± S.D. for three independent experiments with three repetitions.

To determine the necrotic effect of different flavonoids on normal cells, human lymphocytes were induced to proliferate by phytohemagglutinin for 72 h, and the amount of LDH released into the media was measured. The percentage of LDH released after treating the lymphocytes with quercetin, quercetagetin and patuletin was below 8%. Upon comparing this percentage to the amount released by the tumor cells, it was determined that the necrotic effect inside non-tumor cells was not significant. Our results show that treatment with the compounds evaluated—quercetin, quercetagetin and patuletin—did not cause any necrotic effect in the lymphocytes. This result is suggestive of a selective effect of quercetin, quercetagetin and patuletin in non-tumor cells (Figure 13).

## 3. Discussion

Cancer is still considered the most detrimental disease for humans. Extensive research suggests that quercetin may be an antiproliferative agent [24] and that it interacts with DNA [25], causes tumor regression [26] and apoptosis [27]. For this reason, it is important to broaden our knowledge of other compounds derived from natural products, since it is well-known that changes in the general structure of flavonoids function to enhance many biological activities [28]. In terms of their structure, however, the flavonoids quercetin, quercetagetin and patuletin differ only in their substitution patterns in C6, as quercetin has a hydrogen atom, quercetagetin has a hydroxyl group, and patuletin has a methoxy group, at this position. Additionally, all three compounds have a catechol, C2–C3 unsaturation and 3-hydroxyl groups, which have been reported to participate in various biological activities [29]. Biological activity has been attributed to phytochemicals obtained from species of *Tagetes* worldwide [30], including cytotoxic and antiproliferative action [31]. However, before the present study, no one knew if quercetagetin and patuletin molecules were capable of inducing apoptosis and/or necrosis, or if they would also affect normal cells at the same time and in the same models when compared to quercetin [32,33,34,35]. Our study presents interesting evidence which demonstrates that quercetin, quercetagetin and patuletin do indeed have anti-proliferative activity. Moreover, it appears likely that the change in C6 of the flavonoid skeleton of quercetagetin and patuletin could be a potentiator of anti-proliferative activity in SK-Lu-1 cells, as evidenced by comparing the IC_50_’s used in the treatments described.

Our results also show that quercetin and patuletin do not produce a necrotic effect. Quercetagetin was present only in tumor cell lines. These findings suggest that cell elimination is not achieved through necrotic cell death; rather, treating these cancer cell lines with flavonoids evidenced the presence of apoptosis, distinguished by the classic morphological changes of cytoplasmic and nuclear compaction. The presence of active caspase-3, caspase-8, caspase-9 and cleaved PARP in the treated cells lends additional support to this statement. We could speculate that the substitution of the –OH group for the methoxyl group in C6 between quercetagetin and patuletin [21] accounts for this. What has been ascertained is that quercetin is capable of inducing apoptosis [36]. Our work shows that quercetin, quercetagetin and patuletin all cause significant nuclear fragmentation and have a great capacity to induce caspase-3 activation, sometimes even greater than camptothecin, a well-known inducer of apoptosis [37].

Our results clearly show that quercetin, quercetagetin and patuletin all have good cytotoxic activity against different cancer cell lines, and that they achieve cell elimination through the ordered cell-death process called apoptosis. In addition, the results indicate that these compounds induce the intrinsic apoptotic pathway. Also, quercetin and quercetagetin evidenced a selective pro-apoptotic activity against the SK-Lu-1 lung cancer cell line. In this regard, it is important to emphasize that the occurrence of side effects during current chemotherapy treatments are related to the necrotic activity caused by the compound(s) applied.

In addition to demonstrating the pro-apoptotic action exerted by quercetin, quercetagetin and patuletin inside tumor cells, we were also able to determine that these compounds show a selective effect on non-tumor cells, since when normal lymphocytes were treated with the doses applied to the cancer cell lines, the necrotic effect measured was not statistically-significant. Finally, and contrary to their effect on tumor cells, the compounds evaluated herein exhibited a non-antiproliferative effect inside non-tumor cells, relevant data that could be used in other investigations [38] with flavonoids [39]. All these results allow us to propose these flavonoids and modifications in substitution patterns in C6 as options that have selective activity against cancer cells but do not damage normal cells, indicating that they may have future utilization in cancer treatment or other biological activities.

## 4. Materials and Methods

### 4.1. Instrumentation

Melting points were determined using a Fisher Scientific (Pittsburg, PA, USA) apparatus. NMR spectra were recorded on an Advance III 700 MHz spectrometer (Bruker, Billerica, MA, USA) with the residual solvent peak as reference. HR-MS-DART data were obtained by an AccuTOF JMS-T100LC mass spectrometry system (Jeol Ltd., Tokyo, Japan). HPLC-UV analysis were run by an Agilent 1200 Series Binary SL system (Agilent, Carpinteria, CA, USA) equipped with a 2996 UV-Vis diode array detector (Waters Corporation, Milford, MA, USA). The samples were dissolved in methanol and analyzed on a Luna 3 μm C18 (100 × 2.0 mm, 100 Å) column (Phenomenex, Torrance, CA, USA); the mobile phase was composed by water (A) and methanol (B). The elution was performed in gradient conditions, starting from 20 to 100 of B in 30 min; the rate flow was 0.2 mL/min and the UV detection was fixed at 254 nm. The separation of quercetagetin and patuletin were carry out by reverse-phase thin layer chromatography plates (20 × 10 cm, 0.25 mm) (Macherel-Nagel, Düren, Germany). The extract was obtained concentrating in under vacuum using a rotary evaporator (R-100, Büchi, Meierseggstrasse, Switzerland). Quercetin was purchased from Sigma-Aldrich (Sigma, St. Louis, MO, USA).

### 4.2. Plant Materials

Flowers of *Tagetes erecta* and *Tagetes patula* were collected from October–November in Puebla and the State of Mexico; Mexico. Two prepared herbarium specimens were authenticated and deposited in the National Herbarium of Mexico (MEXU), under folio numbers 1409057 and 1409058 respectively.

### 4.3. Extraction and Isolation

Quercetagetin and patuletin were isolated from *Tagetes erecta* and *Tagetes patula* flowers respectively. Once the flowers were cut, we separated the ligules from the receptacles, then, 500 g of each flower were subjected to a static maceration at room temperature in ethanol (1L) for 24 h, the solution was filtered, and ethanol was concentrated in vacuum using a rotary evaporator to give a residue (10 g). The separation of extracts (100 mg) was performed utilizing reverse-phase thin layer chromatography plates (20 × 10 cm, 0.25 mm) eluting with ethyl acetate/methanol/water 7.8:1.2:1.0. This separation conditions were utilized for each extract to afford quercetagetin (70 mg) and patuletin (30 mg).

### 4.4. Compound Characterization

*Quercetagetin* (**2**). Yellow solid; m.p. 255–260 °C; ^1^H-NMR (700 MHz, DMSO-*d*_6_) and ^13^C-NMR (176 MHz, DMSO) see Table 1. HR-DART-MS (positive ion mode) *m*/*z* 319.04553 [M + H]^+^ (Calcd. for C_15_H_11_O_8_: 319.04539); HPLC-UV area peak = 95.17%, retention time = 16.454 min; UV (CH_3_OH) λ_max_ 258.0, 361.7 nm.

*Patuletin* (**3**). Yellow solid, 260–263 °C. ^1^H-NMR (700 MHz, Methanol-*d*_4_) and ^13^C-NMR (175 MHz, Methanol-*d*_4_) see Table 1. HR-DART-MS (positive ion mode) *m*/*z* 333.06114 [M + H]^+^ (Calcd. for C_16_H_13_O_8_: 333.06104); HPLC-UV area peak = 89.47%, retention time = 19.865 min; UV (CH_3_OH) λ_max_ 256.8, 367.1 nm.

### 4.5. Cell Culture

The following cell lines were purchased from the American Type Culture Collection (ATCC Rockville, MD, USA): CaSki, (cervical cancer), MDA-MB-231 (breast cancer), and SK-Lu-1 (lung cancer). They were cultured in RPMI-1640 medium (GIBCO, Invitrogen Corp., Grand Island, NY, USA) containing 5% Newborn Calf Serum (NCS, GIBCO, Invitrogen Corp., Grand Island, NY, USA) with red phenol supplemented by benzylpenicillin. All cultures were stored in a humidified atmosphere with 5% CO_2_ at 37 °C. All cell-based assays were performed using cells in the exponential growth phase.

### 4.6. Cell Proliferation Assay

Assays were performed by seeding 7500 cells/well in 96-well tissue cultured plates in a volume of 100 μL of RPMI-1640 medium supplemented with 5% NCS per well. Cells were allowed to grow for 24 h in the culture medium prior to exposure to the compounds. Also, 1% of vehicle (EtOH) was added to the control cells. Antiproliferative activity (IC_50_) was determined after 24 h by crystal violet staining [40]. Cellular density was determined by measuring absorbance at 590 nm on an Enzyme-linked ImmunoSorbent Assay (ELISA) plate reader (Tecan, Seestrasse, MÄ, Switzerland).

### 4.7. Determination of LDH

Necrotic activity was determined by means of the LDH Cytotoxicity Assay Kit (BioVision, Milpitas, CA, USA). following the manufacturer’s instructions. LDH oxidizes lactate to pyruvate, which then reacts with the tetrazolium salt 2-(4-iodophenyl)-3-(4-nitrophenyl)-5-phenyltetrazolium (INT) to produce formazan. The increase in the amount of formazan generated in the culture supernatant directly correlates to the increase in the number of lysed cells. Formazan dye is water-soluble and can be detected with a spectrophotometer at 500 nm [41].

### 4.8. Observation of Morphological Characteristics of Apoptotic Cells by Fluorescence Microscopy

Cells were cultured in glass coverslips and treated with the IC_50_ of quercetin, quercetagetin and patuletin for 24 h. They were then fixed in 2% paraformaldehyde in PBS at pH 7.2 for 20 min. After rinsing in the same buffer, they were stained with 4,6-diamidino-2-phenylindole (DAPI) and evaluated under an Eclipse E600 microscope (Nikon, Melville, NY, USA). Images were recorded with a DXM1200F digital camera (Nikon, Melville, NY, USA).

### 4.9. CFSE-Labeling Assay

Heparinized blood samples were obtained from healthy human volunteers. Peripheral blood mononuclear cells (PBMCs) were isolated using standard Hypaque (Sigma-Aldrich, St. Louis, MO, USA) gradient density centrifugation. The PBMCs were washed twice with RPMI 1640 medium (GIBCO) containing 10% NCS, penicillin (100 U/mL), and streptomycin (100 U/mL). The lymphocyte population was further enriched (ELP) by eliminating adherent cells (i.e., the cells were incubated at 37 °C, 5% CO_2_ for 1 h, and non-adherent cells were harvested). The ELPs were re-suspended in RPMI-1640 medium at a concentration of 1 × 10^6^ cells/mL and 5(6)-carboxyfluorescein diacetate *N*-succinimidyl ester (CFSE) (Sigma-Aldrich) was added to the cell suspension to a final concentration of 12 μM, and then incubated for 15 min at room temperature in the dark. Labeling was completed by adding the same volume of NCS during 5 min at room temperature to quench the free CFSE. The labeled cells were washed five times with sterile PBS containing 10% NCS, counted, and then re-suspended in RPMI-1640 medium at 1 × 10^6^ cells/mL [42]. Unstimulated, phytohaemagglutinin (PHA)-stimulated, or treated, cells were plated at 2 × 10^5^ cells/mL in 96-well, flat-bottomed cell culture plates, and five replicate samples for each treated amount were prepared. Cells were incubated in a 5% CO_2_ incubator at 37 °C for 72 h. Cultured cells were harvested, washed twice with PBS, fixed with 1% formaldehyde, and then analyzed by flow cytometry to acquire a minimum of 20,000 events from each sample. Data analysis was performed using CellQuest software (Becton–Dickinson, Franklin Lakes, NJ, USA).

### 4.10. Immunolocalization of Active Caspase-3, Active Caspase 8 and Active Caspase-9 by Flow Cytometry

The CaSki, MDA-MB-231 and SK-Lu-1 cells were seeded at 105 cells/mL in 60-mm tissue culture plates and allowed to grow for 24 h in culture medium before being treated with their respective IC_50_. Cells were harvested with versene solution. For active caspase-3, caspase-8 and caspase-9 immunodetection, the cells were fixed and permeabilized in 50% methanol in PBS, washed in PBS, incubated with primary antibody anti-active-caspase-3 (Novous Biologicals, Littleton, CO, USA), anti-active-caspase-8 (Novous Biologicals, Littleton, CO, USA) or anti-active-caspase-9 (Novous Biologicals, Littleton, CO, USA), and then diluted in PBS (1:500) for 18 h. Next, the cells were washed and incubated with the secondary goat anti-rabbit antibody with FITC (SIGMA, St. Louis, MO, USA), and then diluted to 1:200 in PBS for 2 h. The samples were analyzed by flow cytometry, acquiring a minimum of 20,000 events from each sample. Data analysis was performed using CellQuest software (Becton–Dickinson, Franklin Lakes, NJ, USA) [43].

### 4.11. Western Blot Analysis

Protein expression levels were evaluated using western blot assays. The CaSki, MDA-MB-231 and SK-Lu-1 cells were seeded at 105 cells/mL in 60-mm tissue culture plates and allowed to grow for 24 h in culture medium before being treated with their respective IC_50_. Cells were harvested with versene solution. Next, the cells were incubated for 15 min in lysis buffer (50 mMTris–Cl, pH 7.5; 150 mM NaCl, 0.1% SDS, 1 mM PMSF, 0.5% sodium deoxycholate, and 1% Nonidet P-40), supplemented with the complete protease inhibitor cocktail (Roche, Mannheim, Germany). Total proteins were measured by the Qubit system using the Qubit 3 Fluorometer (Thermo Fisher Scientific, Waltham, MA, USA), as follows: 100 µg of total proteins were loaded onto a 12% SDS-PAGE gel before being transferred to polyvinylidene fluoride (PVDF) membranes, which were blotted using the SNAP i.d.^®^ 2.0 Protein Detection System. Briefly: membranes were incubated in blocking buffer, then incubated with anti-active caspase-3 and anti-PARP antibodies at a dilution of 1:5000. After that, the proteins were tagged by incubation with peroxidase-conjugated secondary antibody (Jackson, Newmarket, UK) at 1:10,000 in blocking buffer. Using Horse Radish Peroxidase (HRP) as the substrate (Immobilon Western, Millipore Co., Darmstadt, Germany.), specific labeling was detected by chemiluminescence. Amersham Biosciences Hyperfilm (Amersham Biosciences, Corston, UK) was exposed to the membranes to detect chemiluminescence.

### 4.12. Statistical Analysis

Means and standard deviations (SD) were calculated using Excel (Microsoft Office, 2016 Version, Redmond, WA, USA). Statistical analysis of differences was carried out by analysis of variance (ANOVA) using SPSS 10.0 for Windows (Microsoft, Redmond, WA, USA). A *p* value < 0.05 (Student’s *t* test) was considered significant. In all cases, the data represent three independent experiments performed in triplicate.

## 5. Conclusions

Our conclusion, therefore, is that quercetin quercetagetin and patuletin all demonstrate antiproliferative activity in a dose-dependent manner, with only quercetagetin showing significant necrotic activity. All three compounds induced the intrinsic apoptotic route, but none of them affected the proliferation of normal cells. These results suggest that the presence of a methoxyl group in C6 enhances the potency of flavonols. In future studies, structural modifications will be made as we continue to conduct research on this type of biological activity in an effort to improve our understanding of these compounds and how they could be incorporated into applications to combat different types of cancer in the future.

## Figures and Tables

**Table 1:** ^1^H and ^13^C-NMR data of quercetagetin (**2**) and patuletin (**3**) (ppm).

2 *^a^*			3				
				Measured *^b^*		Literature *^c^*	
Position	δ_c_ (*J* in Hz)	δ_H_ (*J* in Hz)	Position	δ_c_ (*J* in Hz)	δ_H_ (*J* in Hz)	δ_c_ (*J* in Hz)	δ_H_ (*J* in Hz)
4	175.84		4	177.58		177.86	
5	153.58		9	158.46 ^*d*^		158.76 *^d^*	
7	148.81		5	153.61 ^*d*^		153.89 *^d^*	
4′	147.57		7	153.00 ^*d*^		153.28 *^d^*	
2	146.64		4′	148.82		149.10	
6	145.88		2	148.28		148.56	
3′	145.04		3′	146.22		146.51	
3	135.32		3	136.96		137.25	
9	128.53		6	132.17		132.47	
1′	122.21		1′	124.14		124.43	
6′	119.91	7.53 (dd, *J* = 8.5, 2.2)	6′	121.72	7.64 dd (*J* = 8.5, 2.2)	122.02	7.65 dd (*J* = 8.4, 2.2)
5′	115.59	6.88 (d, *J* = 8.5)	2′	116.22	7.74 d (*J* = 2.2)	116.52	7.76 (*J* = 2.2)
2′	115.03	7.66 (d, *J* = 2.2)	5′	116.02	6.89 d (*J* = 8.5)	116.31	6.92 d (*J* = 8.5)
10	103.31		10	104.95		105.25	
8	93.22	6.49 (s)	8	94.70	6.50 s	95.00	6.52 s
			OCH_3_	60.97	3.89 s	61.27	3.92 s
C5-OH		12.25 (s)					
C6-OH		10.48 (s)					
C3′-OH		9.55 (s)					
C4′-OH		9.28 (s)					
C3-OH		9.19 (s)					
C7-OH		8.65 (s)					

*^a^* 700 MHz, DMSO-*d*_6_; *^b^* 700 MHz, CD_3_OD; *^c^* 400 MHz, CD_3_OD; ^*d*^ these values may be interchangeable.

**Table 2:** Antiproliferative activity of the quercetin, quercetagetin and patuletin compounds in tumor cell lines ^1^.

Compound/Cell line	CaSki	MDA-MB-231	SK-LU-1
**Quercetin**	39 (129)	55 (129)	39 (181)
**Quercetagetin**	88 (276)	60 (188)	19 (59)
**Patuletin**	37 (111)	86 (258)	18 (54.17)

^1^ IC_50_ μg/mL (μM).

**Table 3:** Percentage of positive cells to active caspase-3 in cultures of CaSki, MDA-MB-231 and SK-Lu-1 cells treated with IC_50_ values of quercetin, quercetagetin and patuletin.

	CaSki	MDA-MB-231	SK-LU-1
	**Quercetin**		
Control	4	3	4
EtOH	5	3	5
Quercetin	14	37	45
Camptothecin	30	35	60
	**Quercetagetin**		
Control	4	4	4
EtOH	6	5	5
Quercetagetin	31	20	45
Camptothecin	50	25	50
	**Patuletin**		
Control	3	5	3
EtOH	4	5	5
Patuletin	40	43	36
Camptothecin	65	32	43

## Supplementary Materials

The following are available online. Figure S1: ^1^H-NMR spectrum of quercetagetin (**2**), Figure S2: ^13^C-NMR spectrum of quercetagetin (**2**), Figure S3: HSQC spectrum of quercetagetin (**2**), Figure S4: HMBC spectrum of quercetagetin (**2**), Figure S5: HR-DART-MS positive ion mode spectrum of patuletin (**3**), Figure S6: HR-DART-MS positive ion mode spectrum of quercetagetin (**2**), Figure S7: Detection of active caspase-8 in CaSki, MDA-MB-231 and SK-Lu-1 cell cultures exposed to quercetin, quercetagetin and patuletin at the aforementioned IC_50_ values, Figure S8: Detection of active caspase-9 in CaSki, MDA-MB-231 and SK-Lu-1 cell cultures exposed to quercetin, quercetagetin and patuletin at the aforementioned IC_50_ values.
