# Supplementary material for: Quercetagetin and Patuletin: Antiproliferative, Necrotic and Apoptotic Activity in Tumor Cell Lines
Source: Molecules. 2018 Oct 9;23(10):2579. doi: 10.3390/molecules23102579 (PMC6222523; doi:10.3390/molecules23102579)
Supplement: Supplementary file 1 [file molecules-23-02579-s001.pdf]

molecules

MDPI

Quercetagetin and patuletin: antiproliferative, necrotic and apoptotic activity in tumor cell lines

Jesús J. Alvarado-Sansininea [1], Luis Sánchez-Sánchez [2], Hugo López-Muñoz [2], María L. Escobar [3], Fernando Flores-Guzmán [2], Rosario Tavera-Hernández [1] and, Manuel Jiménez-Estrada [1,*]

[1] Laboratorio 2-10. Departamento de Productos Naturales, Instituto de Química, Universidad Nacional Autónoma de México, México. ; manueljemex@gmail.com

[2] Laboratorio 6 3er piso. UMIEZ, Facultad de Estudios Superiores Zaragoza, Universidad Nacional Autónoma de México, México.; luisss@unam.mx

[3] Laboratorio de Microscopía Electrónica, Departamento de Biología Celular, Facultad de Ciencias, Universidad Nacional Autónoma de México, México.

Content

NMR analysis 2

HR-DART-MS analysis... 4

Dot plot Caspase-8...5

Dot plot Caspase-9... 6

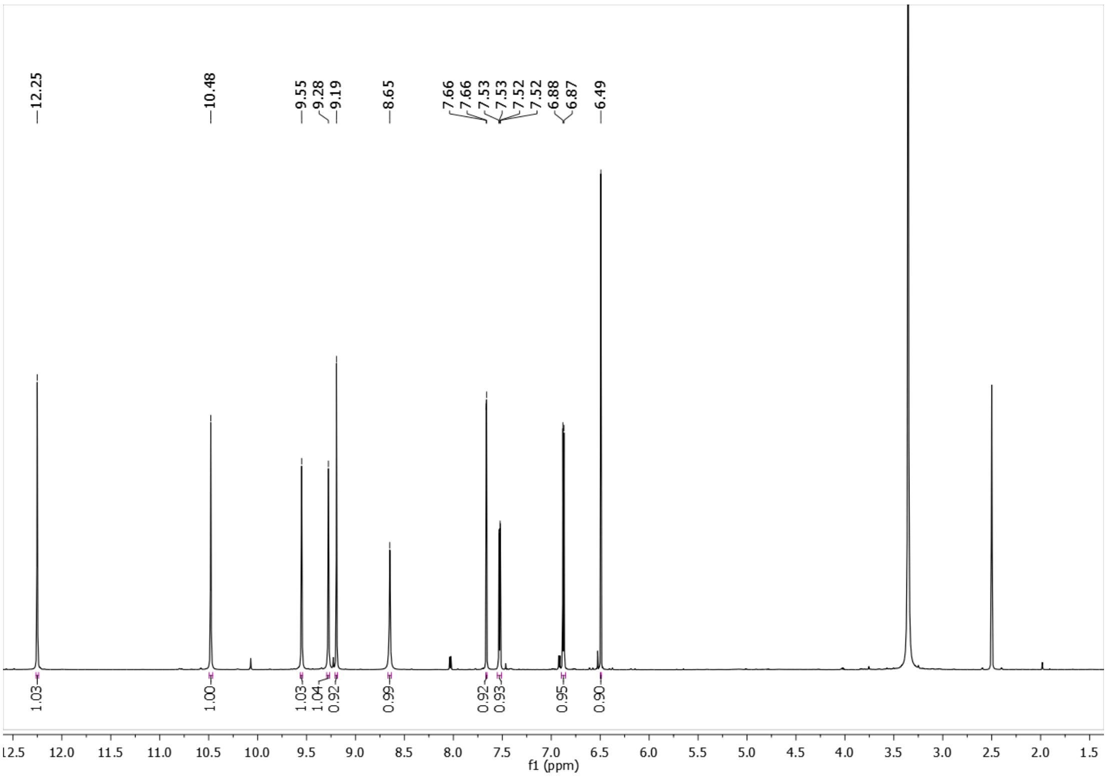

Figure S1. ^{1}H Spectrum of quercetagetin

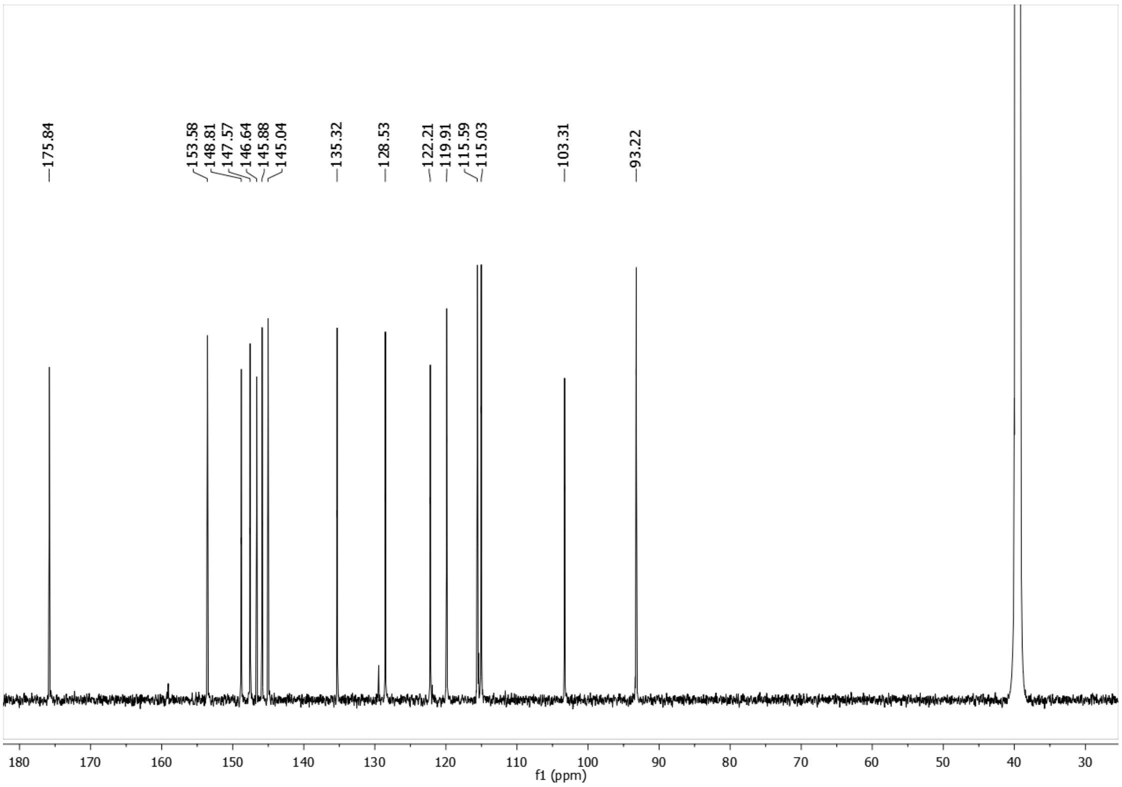

Figure S2. ^{13}C Spectrum of quercetagetin

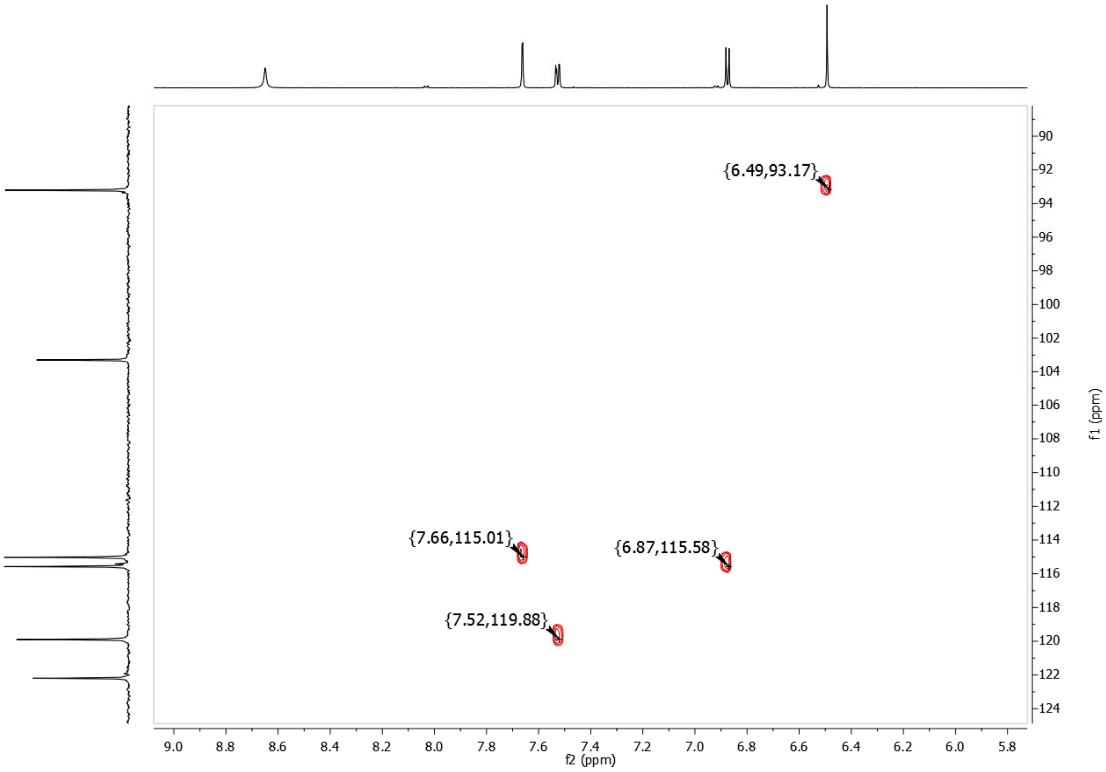

Figure S3. HSQC Spectrum of quercetagetin

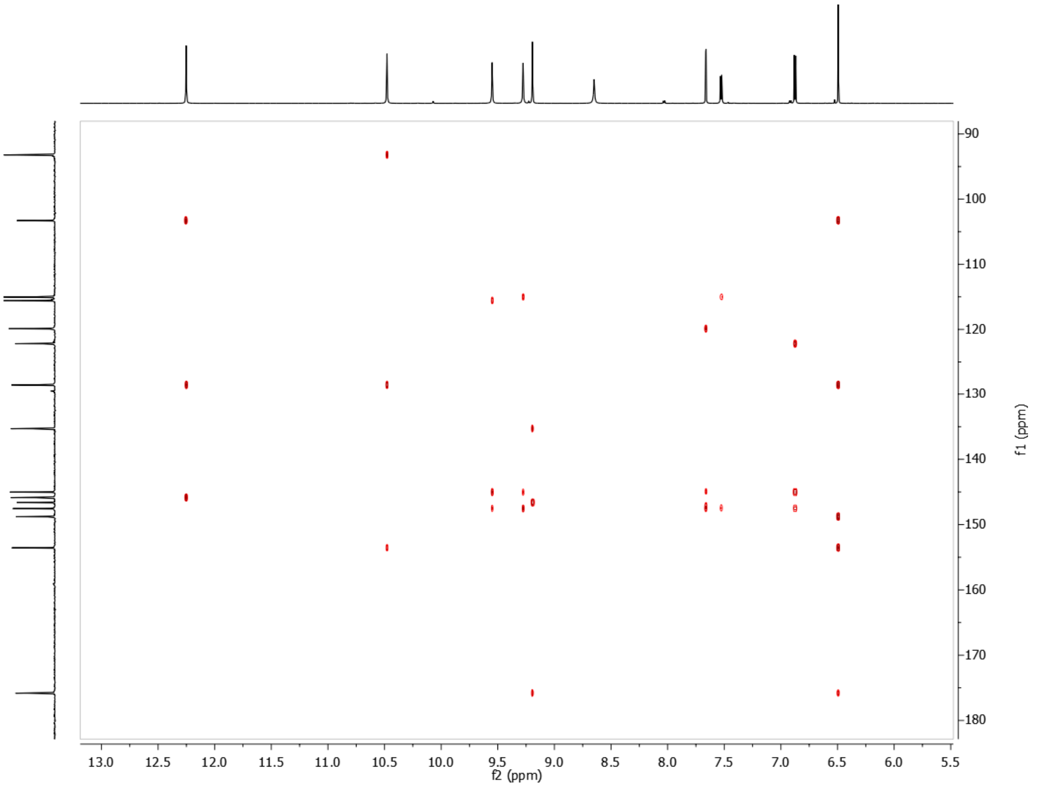

Figure S4. HMBC Spectrum of quercetagetin

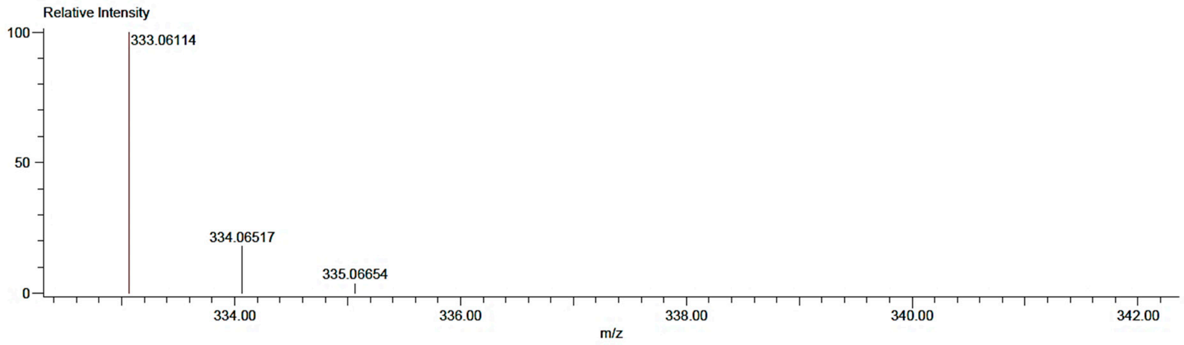

Mass	Intensity	Calc. Mass	Mass Difference (mmu)	Mass Difference (ppm)	Possible Formula	Unsaturation Number
333.06114	379536.22	333.06104	0.10	0.30	$^{12}C_{16}{}^{1}H_{13}{}^{16}O_{8}$	10.5

Figure S5. HR-DART-MS positive ion mode of patuletin

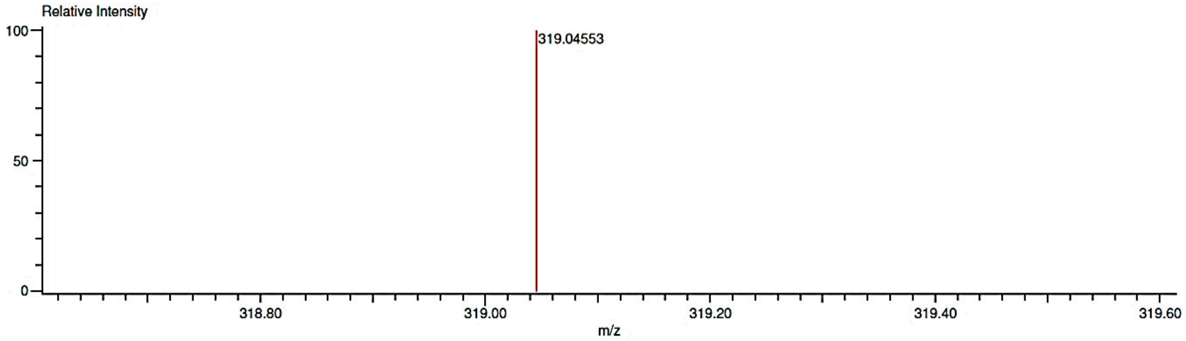

Mass	Intensity	Calc. Mass	Mass Difference (mmu)	Mass Difference (ppm)	Possible Formula	Unsaturation Number
319.04553	734432.75	319.04539	0.13	0.42	$^{12}C_{15}{}^{1}H_{11}{}^{16}O_8$	10.5

Figure S6. HR-DART-MS positive ion mode of quercetagetin

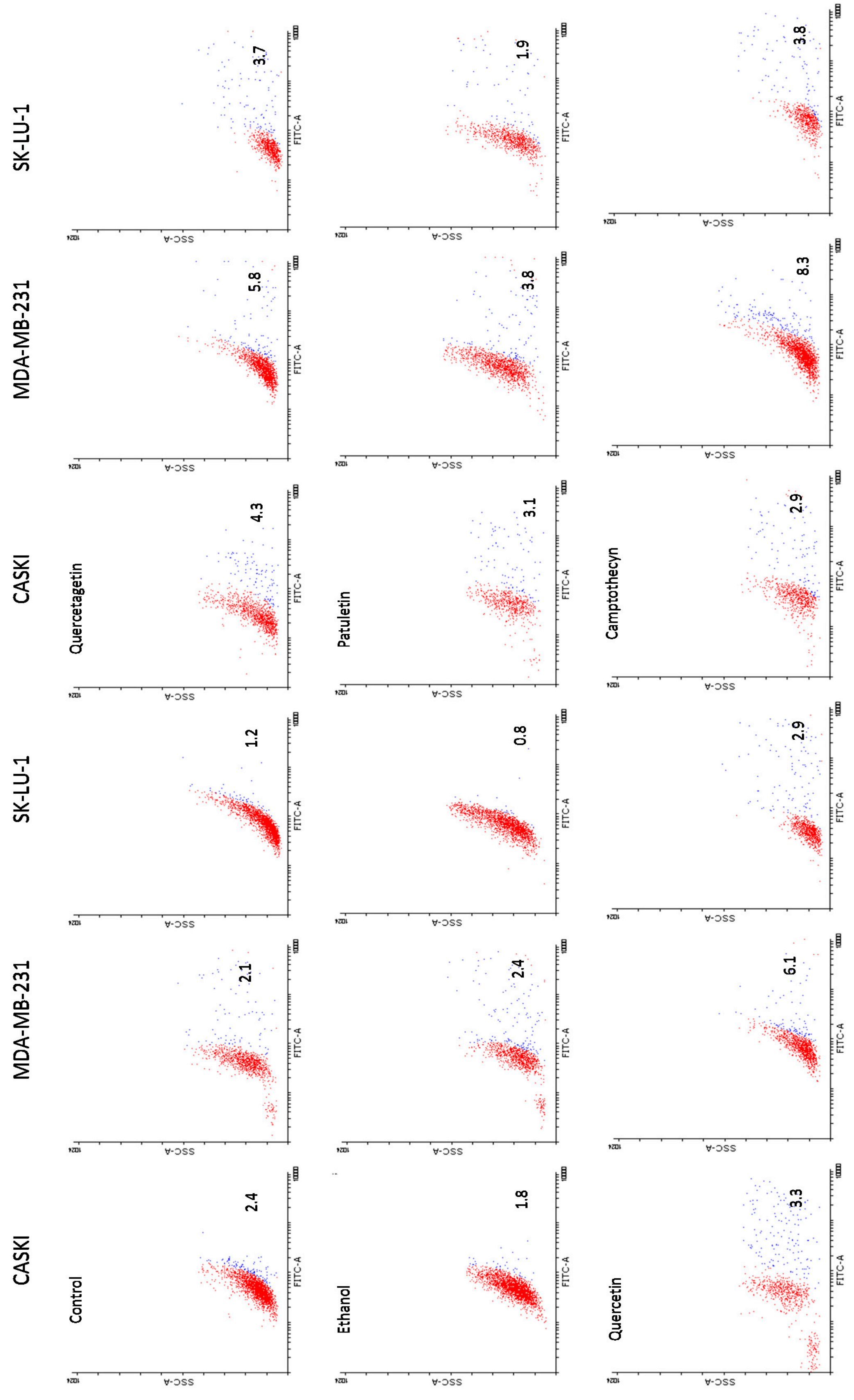

Figure S7. Detection of active caspase-8 in CaSki, MDA-MB-231 and SK-Lu-1 cell cultures exposed to quercetin, quercetagetin and patuletin at the aforementioned IC50 values.

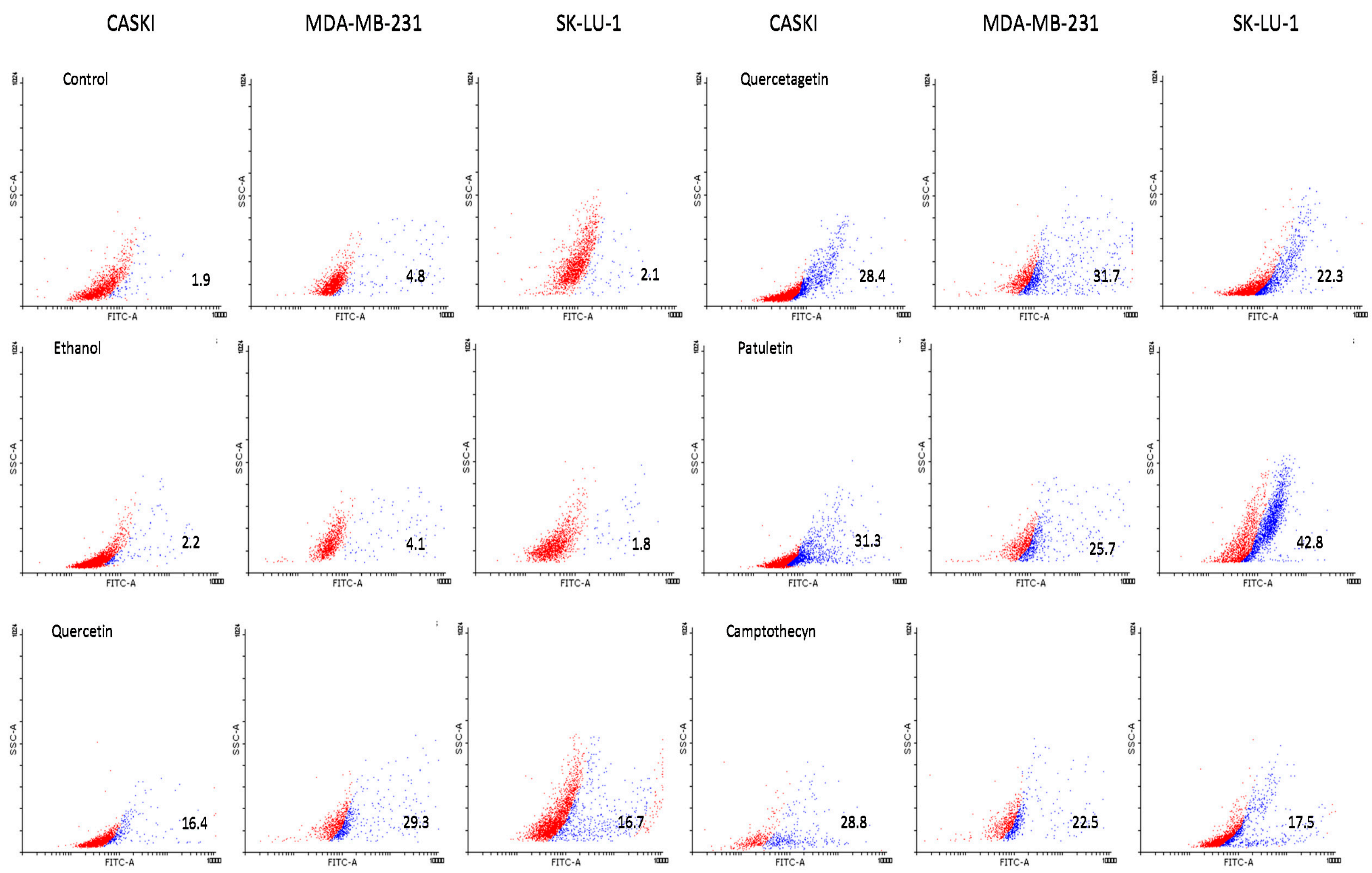

Figure S8. Detection of active caspase-9 in CaSki, MDA-MB-231 and SK-Lu-1 cell cultures exposed to quercetin, quercetagetin and patuletin at the aforementioned IC50 value
